# Simulated microgravity potentiates generation of reactive oxygen species in cells

**DOI:** 10.1007/s41048-016-0029-0

**Published:** 2016-11-07

**Authors:** Fanlei Ran, Lili An, Yingjun Fan, Haiying Hang, Shihua Wang

**Affiliations:** 1 Key Laboratory of Pathogenic Fungi and Mycotoxins of Fujian Province, Key Laboratory of Biopesticide and Chemical Biology of Education Ministry, School of Life Sciences, Fujian Agriculture and Forestry University, Fuzhou, 350002 China; 2 Key Laboratory for Protein and Peptide Pharmaceuticals, Institute of Biophysics, Chinese Academy of Sciences, Beijing, 100101 China

**Keywords:** H_2_O_2_, SMG, ROS production, DNA damage, Apoptosis

## Abstract

Microgravity (MG) and space radiation are two major environmental factors of space environment. Ionizing radiation generates reactive oxygen species (ROS) which plays a key role in radiation-induced DNA damage. Interestingly, simulated microgravity (SMG) also increases ROS production in various cell types. Thus, it is important to detect whether SMG could potentiate ROS production induced by genotoxins including radiation, especially at a minimal level not sufficient to induce detectable ROS. In this study, we treated mouse embryonic stem (MES) cells with H_2_O_2_ and SMG for 24 h. The concentration of H_2_O_2_ used was within 30 μmol/L at which intracellular ROS was the same as that in untreated cells. Exposure of cells to SMG for 24 h did not induce significantly higher levels of intracellular ROS than that of control cells either. Simultaneous exposure of cells to both SMG- and H_2_O_2_-induced ROS and apoptosis in MES cells. Although incubation in medium containing 5 or 30 μmol/L H_2_O_2_ induced a small enhancement of DNA double-strand breaks (DSBs), the addition of SMG treatment dramatically increased DSB levels. Taken together, SMG can significantly potentiate the effects of H_2_O_2_ at a low concentration that induce a small or negligible change in cells on ROS, apoptosis, and DNA damage. The results were discussed in relation to the combined effects of space radiation and MG on human body in this study.

## Introduction

For manned space exploration, it is urgent to investigate the effects of the space environment on human health. Of all the known space environmental factors, microgravity (MG) and space radiation have been recognized as the two major environmental factors. Because of the cost effectiveness and limited access to space flight, simulated microgravity (SMG) on Earth has been widely used in space life research. The integrity of genomic DNA is important for normal physiological functions of cells and DNA damage is related to many diseases such as cancer and aging among others (Lombard et al. 2005; Hoeijmakers 2009). Thus, it is important to investigate the effects of space environment on cellular DNA damage.

It is well known that ionizing radiation (IR) generates reactive oxygen species (ROS) which plays important roles in DNA damage induced by radiation (Tominaga et al. 2004). Interestingly, several lines of evidence showed that SMG increased ROS production in some cell types, such as the PC12 cells, SH-SY5Y cells, and MEF cells (Wang et al. 2009; Qu et al. 2010; Li et al. 2015). It has been reported that SMG delayed the rejoining of double-strand breaks (DSBs) induced by IR and increased the genotoxic effects of IR (Mognato et al. 2009). Mognato et al. also reported that SMG treatment decreased the surviving fraction and increased the *HPRT* mutant frequency in human peripheral blood lymphocytes (Mognato and Celotti 2005). We asked whether SMG could potentiate ROS production and DNA damage induced by space radiation. In the real space environment, space radiation and microgravity act continuously on the body together. Owing to the limitation of the experimental conditions, ionizing radiation and SMG treatment have to be separated into two processes. Thus, in this study, we used H_2_O_2_ instead of radiation and SMG at the same time and investigated whether simulated microgravity could potentiate ROS generation, DNA damage, and apoptosis. Since radiation level inside a space shuttle or a satellite may be too low to induce ROS, we are particularly interested in the following question: when SMG itself cannot induce ROS in a model cell, and the concentration of H_2_O_2_ is kept low so ROS cannot be induced by H_2_O_2_ under 1G, whether SMG can induce ROS in the model cell treated with the low concentration of H_2_O_2_. So far, there have been no reports on the combined effects of SMG and low concentration of H_2_O_2_ on ROS production and DNA damage. In this study, we found that SMG exposure for 24 h or H_2_O_2_ treatment at a concentration below 30 μmol/L for 24 h under 1G could not enhance ROS above untreated mouse embryonic stem (MES) cells, but the combination of these two treatments significantly induced ROS in MES cells. SMG also potentiated the effects of H_2_O_2_ on DNA damage and apoptosis. The results were discussed in relation to the combined effect of space radiation and MG on human body in this study.

## Results

### Combined effects of SMG and H_2_O_2_ on ROS production in wild-type MES cells

**Fig. 1:**
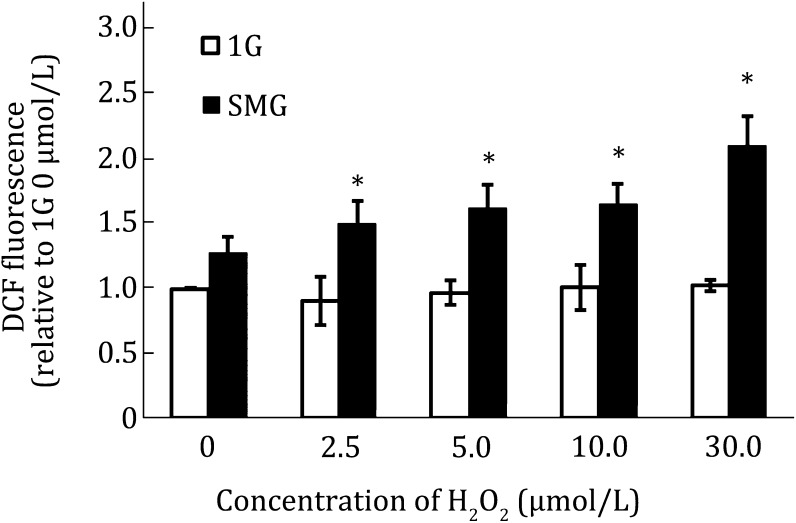
Effects of SMG and H_2_O_2_ treatment on ROS production in wild-type MES cells. Wild-type MES cells were cultured under 1G or SMG for 24 h and treated with H_2_O_2_ at the indicated concentrations at the same time. Then the ROS activity was analyzed with flow cytometry. The experiments were performed thrice independently. The data are shown as mean ± SD. Student’s *t* test, *p < 0.05

To investigate the combined effects of SMG and H_2_O_2_ in ROS production in wild-type MES cells, H_2_O_2_ at the indicated concentrations was added to the media of the cells under 1G and SMG, respectively, and the intracellular ROS level was analyzed by 2′,7′2 dichlorodihydrofluorescein diacetate (DCF-DA) staining. As shown in Fig. 1, the relative DCF fluorescence was slightly higher in the cells cultured under SMG than that in the cells cultured under 1G. However, the difference was not statistically significant. This was consistent with our previous report (Li et al. 2015). In the cells cultured under 1G, treatment of the cells with low concentrations of H_2_O_2_ (from 2.5 to 30 μmol/L) did not alter the intracellular ROS production significantly either. Interestingly, at each indicated concentration of H_2_O_2_, we observed significantly increased intracellular ROS production in the cells cultured under SMG than that in the cells cultured under 1G. These results indicate that SMG triggers ROS production in MES cells incubated in medium containing H_2_O_2_ at the concentration of 30 μmol/L or lower.

### Potentiation of SMG to the effect of H_2_O_2_ on DNA damage

**Fig. 2:**
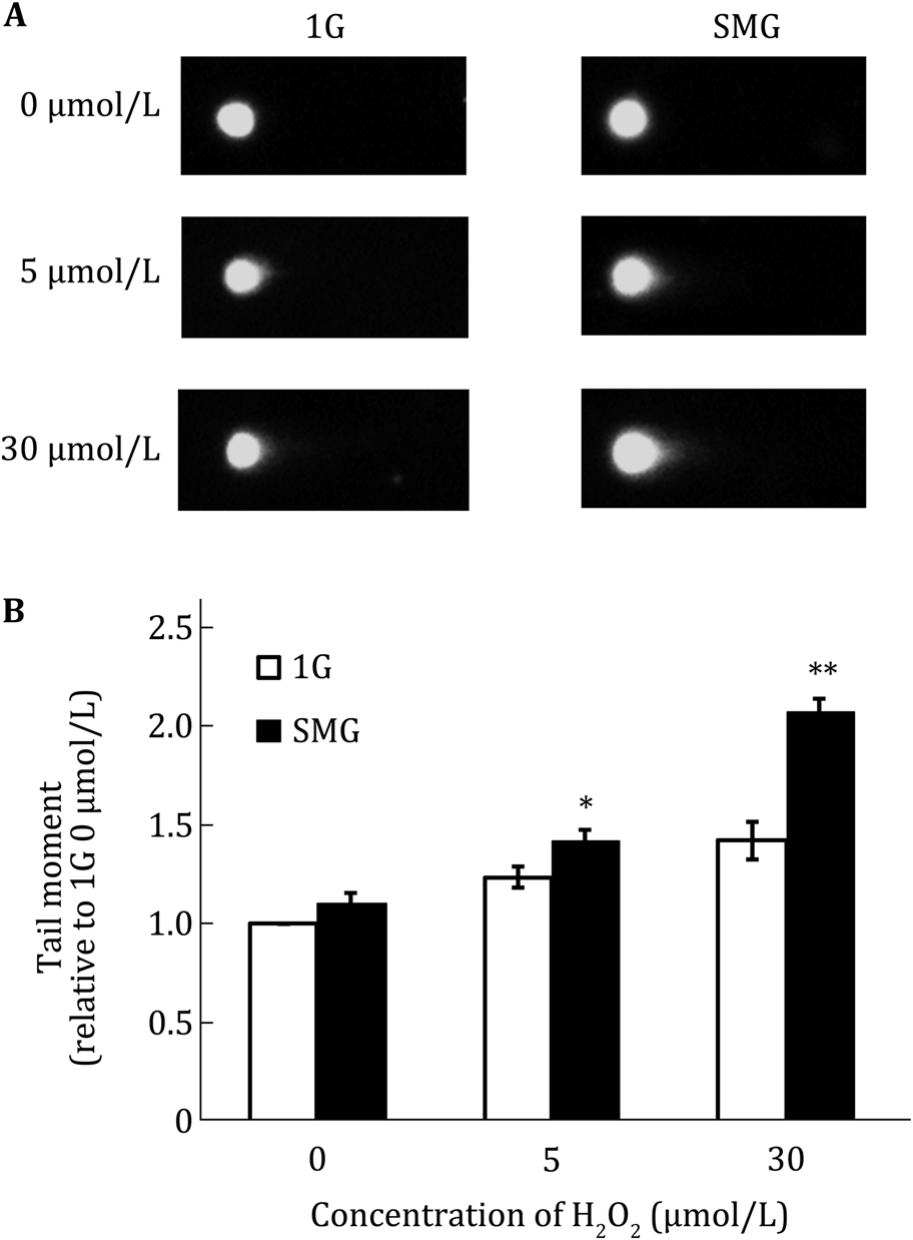
Effects of SMG and H_2_O_2_ treatment on DNA damage in wild-type MES cells. Wild-type MES cells were cultured under 1G or SMG for 24 h and treated with H_2_O_2_ at the indicated concentrations at the same time. Then DNA damage was evaluated using neutral comet assay. The representative results of comet assay are shown in **A** and the quantitative comparison of comet tail moments are shown in **B**. At least 50 cells were scored for analysis of the comet tail moment. The experiments were performed thrice independently. The data are shown as mean ± SD. Student’s *t* test, *p < 0.05, **p < 0.01

ROS can inflict DNA lesions (Schieber and Chandel 2014). To investigate the combined effect of SMG and H_2_O_2_ on DNA damage in MES cells, H_2_O_2_ was added to the media of the cells cultured under SMG and 1G, respectively, at the indicated concentrations, and the DNA damage was analyzed by comet assay. The comet assay is a sensitive method for measuring DNA lesions in single cells. The amount of DNA migration under electric potential indicates the amount of DNA damage in the cell. As shown in Fig. 2, there was no significant difference in DNA damage between the cells cultured under SMG and those cultured under 1G, which was consistent with our previous report (Li et al. 2015). Although 5 or 30 μmol/L H_2_O_2_ did not enhance intracellular ROS levels, it was able to cause higher levels of DNA damage under 1G (Fig. 2B). This elevated level of DNA lesions was small but statistically significant (data not shown). In contrast, when treated with 5 or 30 μmol/L H_2_O_2_, the relative tail moment of the cells cultured under SMG was significantly higher than that cultured under 1G. These results indicate that SMG potentiates the effect of H_2_O_2_ on DNA damage.

### NAC significantly suppresses DNA damage in MES cells treated with both SMG and H_2_O_2_

**Fig. 3:**
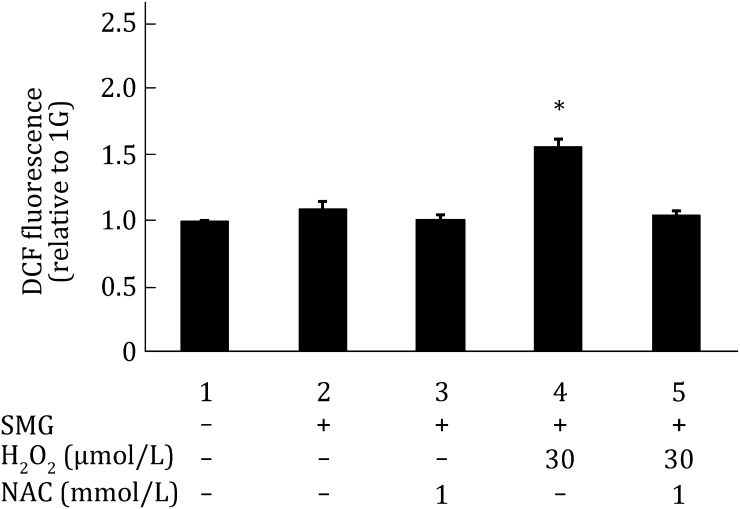
NAC significantly reduces ROS production in MES cells treated with both SMG and H_2_O_2_. Flow cytometric analysis of ROS activity in wild-type MES cells.* Column 1* wild-type MES cells cultured under 1G without any treatment;* Column 2* wild-type MES cells cultured under SMG without any treatment;* Column 3* wild-type MES cells maintained under SMG and treated with 1 mmol/L NAC;* Column 4* wild-type MES cells cultured under SMG and treated with 30 μmol/L H_2_O_2_;* Column 5* wild-type MES cells cultured under SMG and treated with 30 μmol/L H_2_O_2_ as well as 1 mmol/L NAC. The experiments were performed thrice independently. The data are shown as mean ± SD. Student’s *t* test, *p < 0.05 compared with* Column 1*

**Fig. 4:**
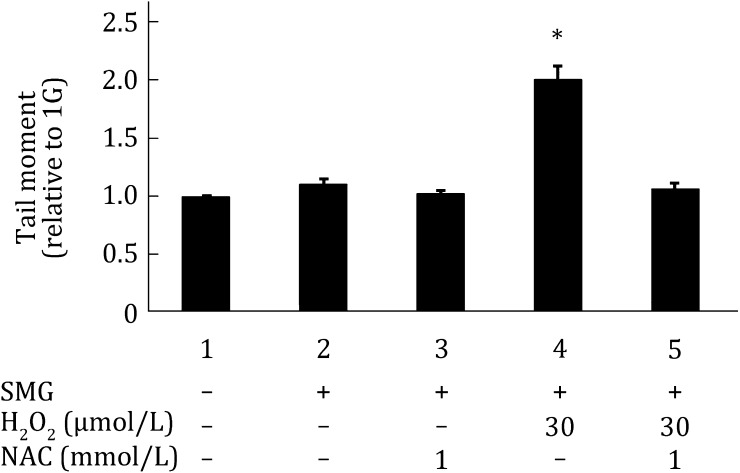
NAC significantly reduces DNA damage in MES cells treated with both SMG and H_2_O_2_. DNA damage was assayed by neutral comet assay in wild-type MES cells.* Column 1* wild-type MES cells cultured under 1G without any treatment;* Column 2* wild-type MES cells cultured under SMG without any treatment;* Column 3* wild-type MES cells cultured under SMG and treated with 1 mmol/L NAC;* Column 4* wild-type MES cells cultured under SMG and treated with 30 μmol/L H_2_O_2_;* Column 5* wild-type MES cells cultured under SMG and treated with 30 μmol/L H_2_O_2_ as well as 1 mmol/L NAC. The experiments were performed thrice independently. The data are shown as mean ± SD. Student’s *t* test, *p < 0.05 compared with* Column 1*

N-acetylcysteine (NAC) is a widely used ROS scavenger (Dhouib et al. 2016). As shown in Fig. 3, 1 mmol/L NAC effectively suppressed ROS induced by the combined treatments of H_2_O_2_ and SMG. 1 mmol/L NAC also effectively suppressed DNA damage induced by the combined treatments of H_2_O_2_ and SMG (Fig. 4), suggesting that the DNA lesions inflicted by the combined treatments of H_2_O_2_ and SMG are mediated by ROS production in cells.

### NAC significantly reduces apoptosis in MES cells treated with both SMG and H_2_O_2_

**Fig. 5:**
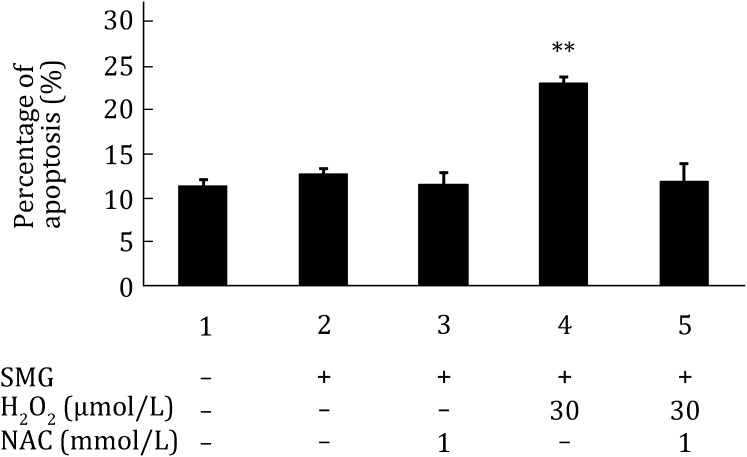
NAC significantly reduces apoptosis in MES cells treated with both SMG and H_2_O_2_. Flow cytometric analysis of apoptosis in wild-type MES cells.* Column 1* wild-type MES cells cultured under 1G without any treatment;* Column 2* wild-type MES cells cultured under SMG without any treatment;* Column 3* wild-type MES cells cultured under SMG and treated with 1 mmol/L NAC;* Column 4* wild-type MES cells cultured under SMG and treated with 30 μmol/L H_2_O_2_;* Column 5* wild-type MES cells cultured under SMG and treated with 30 μmol/L H_2_O_2_ as well as 1 mmol/L NAC. The experiments were performed thrice independently. The data are shown as mean ± SD. Student’s *t* test, **p < 0.01 compared with* Column 1*

As shown above, SMG potentiated the effect of H_2_O_2_ on DNA damage in MES cells, and DNA damage leads to apoptosis (Zhang et al. 2016). Previously we observed that SMG itself was unable to induce apoptosis in wild-type MES cells (Li et al. 2015).We asked whether SMG could potentiate the effect of H_2_O_2_ on apoptosis in MES cells. As shown in Fig. 5, SMG triggered apoptosis in MES cells treated with 30 mmol/L H_2_O_2_ (Fig. 5). NAC treatment effectively reversed the increased apoptosis. Our results indicate that enhanced ROS mediates apoptosis induced by the combined treatments of H_2_O_2_ and SMG.

## Discussion

In this study, we found that SMG exposure alone or H_2_O_2_ treatment at a low concentration alone could not enhance ROS production in MES cells. However, the combination of these two treatments significantly induced ROS production (Fig. 2). SMG also potentiated the effects of H_2_O_2_ on DNA damage and apoptosis. Furthermore, ROS scavenger NAC could inverse these effects in MES cells treated with both SMG and H_2_O_2_ (Figs. 4 and 5).

In mammalian cells, there is a balance of ROS production and scavenging (Aon et al. 2010). A small increase in ROS levels only activates signaling pathways to initiate biological processes, but high levels of ROS also result in damage to DNA, protein, or lipids (Schieber and Chandel 2014). In this study, treatment of the MES cells cultured under 1G with 30 μmol/L H_2_O_2_ did not significantly alter the intracellular ROS production. Consistently, we did not observe increased apoptosis in these cells. It seems that cultured under 1G, MES cells could effectively scavenge the increased ROS induced by 30 μmol/L H_2_O_2_, avoiding the damage of the oxidative stress to the cells. Although 5 or 30 μmol/L H_2_O_2_ did not enhance intracellular ROS levels, it was able to cause slightly but also significantly higher levels of DNA damage than that under 1G (Fig. 2B). It seems that H_2_O_2_ as low as 5 μmol/L could induce increased DNA damage before it was scavenged by the cells.

Wang et al. reported that SMG increased the amount of ROS in rat PC12 cells (Wang et al. 2009). Previously, we observed significant SMG-induced ROS production and DNA damage in *Rad9* ^−/−^ MES but not in wild-type MES cells (Li et al. 2015). In this study, SMG treatment potentiated 30 μmol/L H_2_O_2-_induced ROS production, as well as DNA damage and apoptosis in wild-type MES cells, indicating the synergistic effects of SMG and H_2_O_2_. Altogether, these results indicate that SMG is a weak genotoxic stress and could break the balance of ROS production and scavenging under the stress of low dose of H_2_O_2_. However, the precise mechanisms need further investigation.

In addition to microgravity, space radiation is another key detrimental factor in space environment. It has been reported that the superoxide increased by 16.5% after 15 min of 5 cGy radiation in A549 cells (Chen et al. 2015). Manganese superoxide dismutase (SOD_2_) could be induced by IR with the dose as low as 2 cGy (Veeraraghavan et al. 2011). To our knowledge, there is no report on ROS production induced by IR at the doses lower than 2 cGy. It should be noted that at orbital altitudes near that of the International Space Station, the dose-equivalent to the astronauts is about 0.3 Sv per year (an absorbed dose of 1 Gy by alpha particles will lead to an equivalent dose of 20 Sv) (Thirsk et al. 2009), which means the effects of space radiation alone on ROS production may be not significant. However, we have shown that SMG potentiated ROS production induced by low concentrations of H_2_O_2_. Thus in the real space environment, microgravity may also potentiate space radiation-induced ROS production and DNA damage, which should be tested in the real space experiments. Furthermore, during manned space travel, the astronauts experience stressful conditions such as loneliness, tension, and lack of exercise. These may also lead to increased ROS levels in the astronauts. Thus whether SMG potentiates ROS production induced by these conditions deserves further investigation.

NAC is widely used in ROS scavenging (Dhouib et al. 2016). Here, we found that NAC could effectively suppress SMG and H_2_O_2_-induced ROS production, DNA damage as well as apoptosis in MES cells. Wang et al. also reported the protective effects of NAC on ROS production and cell senescence under SMG treatment (Wang et al. 2009). Qu et al. showed that antioxidants, isorhamnetin and luteolin, could protect neuroblastoma SH-SY5Y cells against microgravity-induced oxidative stress (Qu et al. 2010). These results indicate that antioxidants such as NAC might be used in the protection of ROS stress induced by the combined effects of SMG and other factors of space environment, which may provide valuable strategy for health protection in manned space exploration.

## Materials and methods

### 3D-clinostat

The 3D-clinostat which was used for SMG treatment was provided by Center for Space Science and Applied Research, Chinese Academy of Sciences (Jiang et al. 2008). By employing simultaneous rotations on two axes, the 3D-clinostat can produce an environment with an average of 10^−3^ G, thus simulating microgravity conditions.

### Cell culture

MES cells, originally derived from Joyner’s laboratory (Auerbach et al. 2000), were cultured on gelatin-coated flasks in standard ES cell medium with leukemia inhibitory factor (LIF) according to Joyner AL without a feeder layer (Matise et al. 2000). Cells were seeded in culture flasks (Becton–Dickinson) and were maintained under 1G for 18 h so that the cells could adhere to the flasks. Then the flasks were filled with fresh medium and the air bubbles were eliminated in order to diminish turbulence and shear forces. The 3D-clinostat was placed in an incubator with an atmosphere of 95% air/5% CO_2_ at 37 °C. The day on which the flasks were placed on the clinostat was designated as Day 0. We did not change the culture medium during the experimental period.

The MES cells maintained under 1G or SMG were treated with H_2_O_2_ at the concentrations of 0, 2.5, 5, 10, and 30 μmol/L for 24 h. For antioxidant treatment, the cells were also treated with ROS scavenger NAC (1 mmol/L) for 24 h.

### Apoptosis assays

MES cells were seeded at 5 × 10^5^ cells per 25 cm^2^ culture flask. After treatment, the cells were trypsinized with 0.1% trypsin at 37 °C (Sigma), then washed twice with cold PBS, and resuspended in 1× binding buffer at 1 × 10^6^ cells/mL. After that, the cells were stained with Alexa Fluor^®^ 488 annexin V and PI (Invitrogen) at room temperature for 15 min for flow cytometric analysis.

### Comet assay

Comet assay was performed according to the protocol of Singh et al. (1988) with minor modifications. Firstly, we pre-coated the slides with a thin layer of 1% normal melting agarose. Secondly, the cells were harvested and resuspended at a concentration of 5 × 10^5^ cells/mL. 20 μL of each suspension was added to 80 μL of pre-melted 0.75% low melting agarose and the contents were pipetted onto the pre-coated slide. Thirdly, the slides were immersed in neutral lysis solution in the dark at 4 °C for 2 h. For unwinding of the DNA, the slides were immersed in 1 × TBE buffer in the dark at 4 °C for 30 min. After that, the slides were exposed to ~0.74 V/cm for 25 min in the horizontal electrophoresis chamber. Following electrophoresis, we stained the slides with propidium iodine (PI). Fluorescence images were viewed with a microscope and analyzed by CASP-1.2.2 software (University of Wroclaw).

### ROS activity assays

The cells were stained with 20 μmol/L 2′,7′dichlorodihydrofluorescein diacetate (DCF-DA) (Sigma, USA), and intracellular ROS activity was examined (Shen et al. 2001). The fluorogenic probe DCF-DA is cell-permeable. It diffuses into cells and is deacetylated into the non-fluorescent DCFH by cellular esterases. While, in the presence of ROS, DCFH is rapidly oxidized to highly fluorescent DCF. The fluorescence intensity was measured by flow cytometry (FACSCalibur, Becton–Dickinson, USA) with excitation settings of 488 nm and emission settings of 530 nm, respectively.

### Statistical analysis

The data are shown as mean ± SD. The statistical significance of the difference was analyzed by the Student’s *t* test. *p* < 0.05 was considered statistically significant.

## Abbreviations

MG: Microgravity
IR: Ionizing radiation
ROS: Radical oxygen species
SMG: Simulated microgravity
MES: Mouse embryonic stem
DSB: Double-strand breaks
DCF-DA: 2′,7′2 dichlorodihydrofluorescein diacetate
NAC: N-acetylcysteine
SOD_2_: Manganese superoxide dismutase
LIF: Leukemia inhibitory factor
PI: Propidium iodine
